# Supplementary material for: Estimating dispensable content in the human interactome
Source: Nat Commun. 2019 Jul 19;10:3205. doi: 10.1038/s41467-019-11180-2 (PMC6642175; doi:10.1038/s41467-019-11180-2)
Supplement: Supplementary file 1 — Supplementary Information [file 41467_2019_11180_MOESM1_ESM.pdf]

Estimating dispensable content in the human interactome

Supplementary Information

Ghadie *et al.*

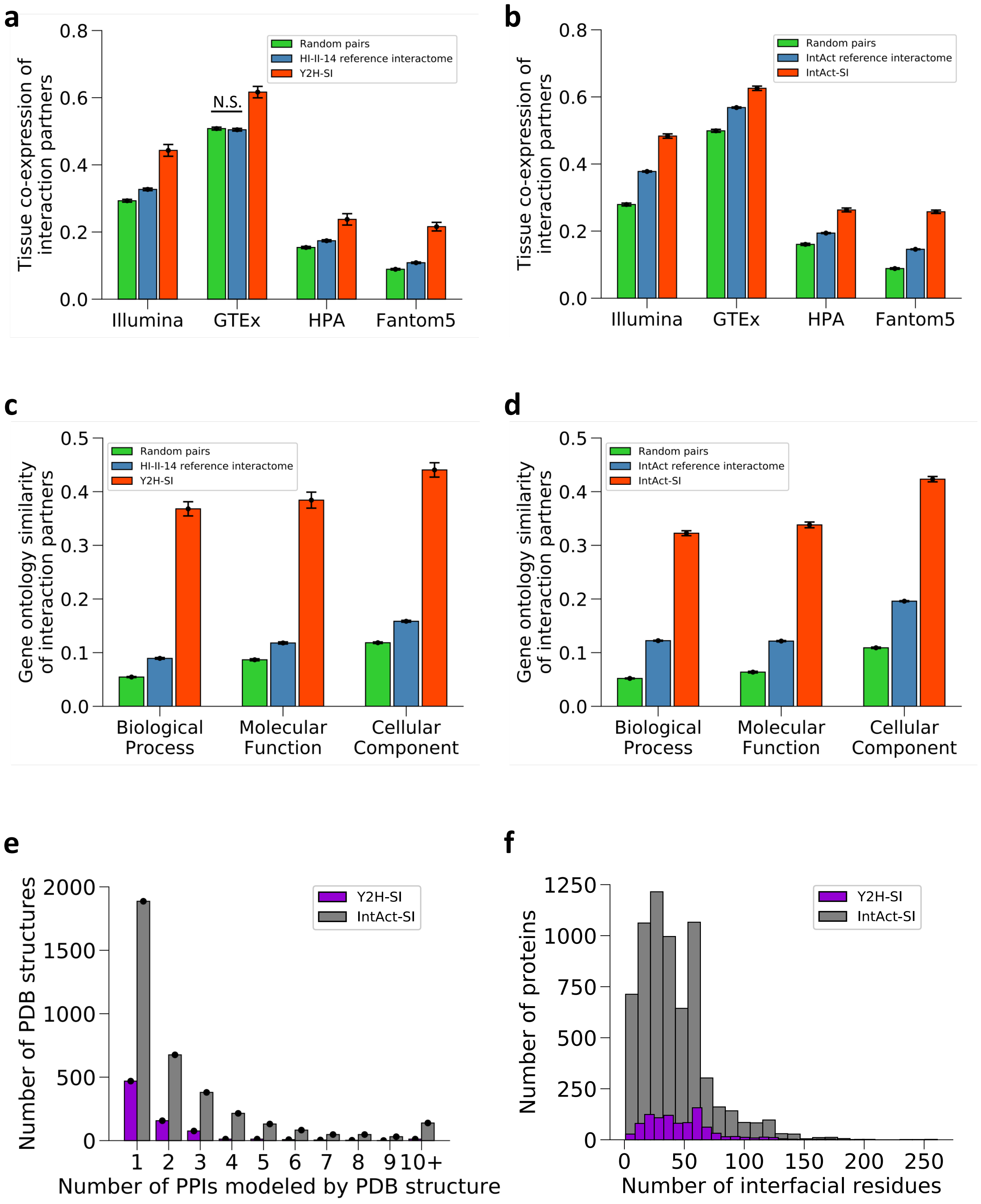

Supplementary Figure 1. Functional and structural description of the human structural interactome

(a) and (b) Tissue co-expression of protein interaction partners in the human structural interactomes (Y2H-SI and IntAct-SI) compared to interaction partners in the human reference

interactomes (HI-II-14 and IntAct) as well as 10,000 randomly selected protein pairs. Error bars represent standard errors of the mean. Unless labeled with N.S. (not significant), all differences between adjacent bars are statistically significant ($p \leq 10^{-4}$, two-sided bootstrap test with 100,000 re-samplings).

(c) and (d) Gene ontology similarity of protein interaction partners in the human structural interactomes (Y2H-SI and IntAct-SI) compared to interaction partners in the human reference interactomes (HI-II-14 and IntAct) as well as 10,000 randomly selected protein pairs. Error bars represent standard errors of the mean. All differences between adjacent bars are statistically significant ($p < 10^{-5}$, two-sided bootstrap test with 100,000 re-samplings).

(e) Distribution of the number of PPIs modeled per PDB structure in the human structural interactome.

(f) Distribution of the number of interfacial residues per protein in the human structural interactome.
