# Supplementary material for: Estimating dispensable content in the human interactome
Source: Nat Commun. 2019 Jul 19;10:3205. doi: 10.1038/s41467-019-11180-2 (PMC6642175; doi:10.1038/s41467-019-11180-2)
Supplement: Supplementary file 3 — Description of Additional Supplementary Files [file 41467_2019_11180_MOESM3_ESM.pdf]

Description of Additional Supplementary Files

File Name: Supplementary Data 1
Description: **PPIs with aligned PDB chain pairs**
PPIs in the human reference interactome with at least one pair of chains in the same PDB structure jointly aligned with significant E-value ($<10^{-10}$).
(A) HI-II-14 interactome
(B) IntAct interactome

File Name: Supplementary Data 2
Description: **Human structural interactome with resolved PPI interfaces**
(A) HI-II-14 structural interactome (Y2H-SI)
(B) IntAct structural interactome (IntAct-SI)

File Name: Supplementary Data 3
Description: **Mutations with geometry-based edgotype predictions**
(A) Non-disease mutations in Y2H-SI
(B) Disease mutations in Y2H-SI
(C) Non-disease mutations in IntAct-SI
(D) Disease mutations in IntAct-SI

File Name: Supplementary Data 4
Description: **Change in PPI binding free energy calculated using BindProfX**
Change in binding free energy ($\Delta\Delta G$) of PPIs upon mutation at the interface, calculated on PPI structural models using BindProfX.
(A) PPIs with non-disease mutations in Y2H-SI
(B) PPIs with disease mutations in Y2H-SI
(C) PPIs with non-disease mutations in IntAct-SI
(D) PPIs with disease mutations in IntAct-SI

File Name: Supplementary Data 5
Description: **Mutations with physics-based edgotype predictions**
(A) Non-disease mutations in Y2H-SI
(B) Disease mutations in Y2H-SI
(C) Non-disease mutations in IntAct-SI
(D) Disease mutations in IntAct-SI

File Name: Supplementary Data 6
Description: **Change in PPI binding free energy calculated using FoldX**
Change in binding free energy ($\Delta\Delta G$) of PPIs upon mutation at the interface, calculated on PPI structural models using FoldX.
(A) PPIs with non-disease mutations in Y2H-SI
(B) PPIs with disease mutations in Y2H-SI
(C) PPIs with non-disease mutations in IntAct-SI
(D) PPIs with disease mutations in IntAct-SI
